# Chondrogenic Maturation Governs hMSC Mechanoresponsiveness to Dynamic Compression

**DOI:** 10.3390/bioengineering12101075

**Published:** 2025-10-03

**Authors:** Farhad Chariyev-Prinz, Ross Burdis, Daniel J. Kelly

**Affiliations:** 1 Trinity Centre for Biomedical Engineering, Trinity Biomedical Sciences Institute, Trinity College Dublin, D02 R590 Dublin, Ireland; 2 Department of Mechanical and Manufacturing Engineering, School of Engineering, Trinity College Dublin, D02 PN40 Dublin, Ireland; 3 Advanced Materials and Bioengineering Research Centre (AMBER), Royal College of Surgeons in Ireland, D02 YN77 Dublin, Ireland; 4 Advanced Materials and Bioengineering Research Centre (AMBER), Trinity College Dublin, D02 PN40 Dublin, Ireland

**Keywords:** dynamic compression, chondrogenesis, tissue engineering

## Abstract

Dynamic compression (DC) bioreactors are widely used to mimic joint loading and study how human mesenchymal stem cells (hMSCs) respond to mechanical cues. However, it remains unclear whether DC alone is sufficient to induce chondrogenesis or how such cues interact during construct maturation. In this study, hMSCs were encapsulated in fibrin hydrogels at different cell densities and subjected to DC without, during, or after TGF-β3-mediated chondrogenic induction. DC alone modestly increased *SOX9* expression but failed to upregulate key cartilage matrix genes such as *ACAN* and *COL2A1*, indicating that mechanical stimulation alone is insufficient to initiate chondrogenesis. When mechanical stimulation was coupled with TGF-β3, a more mature chondrogenic phenotype was observed for high cell seeding densities (HD). To simulate a post-implantation scenario, we applied DC following growth factor withdrawal and observed marked downregulation of *SOX9*, *ACAN*, and *COL2A1* in low-density (LD) constructs. This reduction was not observed in HD constructs, which maintained a more stable chondrogenic phenotype under loading. These findings show that construct maturation critically influences mechanoresponsiveness and suggest that immature grafts may not tolerate mechanical stimulation. DC bioreactors may therefore serve not only to support cartilage engineering but also to predict in vivo graft performance.

## 1. Introduction

Human mesenchymal stem/stromal cells (hMSCs) are widely used to engineer different musculoskeletal tissues [1]. As chondrocytes tend to lose their chondrogenic potential with age and standard in vitro expansion [2,3], hMSCs are increasingly being used as an alternative cell type for tissue engineering (TE) of articular cartilage grafts [4]. When combined with appropriate growth factors and 3D scaffolds, MSCs have been shown to express cartilage-specific extracellular matrix (ECM) components and produce hyaline cartilage-like tissues rich in glycosaminoglycans (GAGs) and type II collagen [1,5,6]. However, despite such encouraging results, the generation of phenotypically stable hyaline cartilage using mesenchymal stem cells (MSCs) remains a challenge, often resulting in the development of fibrocartilage in vitro and endochondral bone formation in vivo [7,8,9]. To tackle these challenges various approaches have been investigated, including co-culture strategies, growth factor priming, exposure to hypoxia as well as mechanical stimulation using bioreactors [9,10,11,12,13]. Nevertheless, the robust and consistent generation of functional hMSC-derived hyaline-like tissue in vitro remains a challenge.

Mechanical cues play a crucial role during development, orchestrating the appropriate and timely formation of different organs and structures [14]. For example, joint cavity formation is impaired when limbs are immobilized [15], whereas the presence of uniaxial dynamic compression (DC) has been shown to promote epiphyseal growth [16]. Additionally, mechanical stimulation contributes to the maintenance of tissue integrity and moderate exercise has been linked to increased sulfated glycosaminoglycans (sGAG) deposition in individuals with increased risk of OA [17,18]. More importantly, mechanical stimulation has also been shown to promote cartilage formation during fracture healing [19,20,21] and facilitate regeneration in animal models [22,23,24]. These observations collectively highlight the pivotal role of biomechanical signals in both development and repair. Various bioreactors have been developed to recapitulate aspects of synovial joint loading in vitro. In this context, DC bioreactors are utilized to emulate the compressive forces present in vivo [25]. Typically, cells of distinct densities are seeded onto 3D scaffolds and then subjected to cyclic uniaxial compression to study the influence of such mechanical cues on MSC chondrogenesis [26,27]. Studies have explored the combinatorial effect of DC and growth factors like TGF-β3 on hMSC chondrogenesis. Although delayed application of DC during culture is generally considered more beneficial for matrix deposition and phenotypic stability, some studies suggest early loading may also be effective [10,28,29,30]. While DC has been implicated in the suppression of hypertrophic differentiation in some contexts, these effects are not consistently observed across studies [10,11,31,32]. These divergent findings underscore the need for more standardized experimental approaches and highlight the importance of considering variables such as scaffold composition, timing of stimulation, and cell density [33].

Another underexplored aspect of cartilage TE involves the transition of engineered grafts from controlled in vitro environments to the mechanically demanding in vivo joint space. In preclinical and clinical scenarios, constructs are typically cultured under supraphysiological growth factor concentrations and are then implanted into environments where such factors are absent or present at much lower concentrations but mechanical loading is prevalent [34,35]. Little is known about how the removal of potent chondrogenic inducers affects the mechanoresponsiveness of hMSCs or whether graft maturity at the time of implantation alters this response. We hypothesized that fundamental culture parameters, including cell density and stimulation timing, can be leveraged to modulate graft maturity and assess its time dependent mechanoresponsiveness.

The goal of this study was to investigate how hMSC chondrogenic maturation influences their responsiveness to DC in 3D fibrin hydrogels. To achieve this, we investigated (1) if DC can initiate chondrogenesis in the absence of TGF-β3; (2) how DC and TGF-β3 interact to regulate chondrogenesis in hydrogels seeded with different hMSC densities, and (3) how the removal of this growth factor after the initiation of chondrogenesis affects engineered graft responses to DC. These findings aim to better inform preconditioning strategies and improve prediction of engineered cartilage performance in vivo.

## 2. Materials and Methods

### 2.1. Cell Isolation and Expansion

Adult human bone marrow-derived mesenchymal stem cells (hBMSCs) were isolated from bone marrow aspirates (1× male, 2× female, Lonza) and expanded in high-glucose Dulbecco’s Modified Eagle Medium (DMEM) GlutaMAX, supplemented with 10% (*v*/*v*) fetal bovine serum (FBS), 100 U/mL penicillin, 100 µg/mL streptomycin (Gibco, Waltham, MA, USA), and 5 ng/mL human fibroblast growth factor-2 (FGF-2; PeproTech, Cranbury, NJ, USA). Cells were cultured at 37 °C in a humidified incubator with 5% CO_2_ and 5% O_2_. Expansion was initiated at a seeding density of 5000 cells/cm^2^ in T175 flasks, and cells were passaged upon reaching 80% confluency. Unless otherwise stated, all experiments were performed using passage 4 cells. Female donor #2 was used in all single donor studies.

### 2.2. Preparation and Culture of Cellular Fibrin Hydrogels

Fibrinogen (Sigma-Aldrich, Saint Louis, MO, USA) was dissolved in a NaCl–aprotinin solution (19 mg/mL NaCl; Sigma-Aldrich; aprotinin from Nordic Pharma, Paris, France) to obtain a final fibrinogen concentration of 50 mg/mL. Thrombin stock was diluted in DMEM containing 40 mM CaCl_2_ at pH 7.2 to yield a final concentration of 5 U/mL (Sigma-Aldrich). To form cellular fibrin hydrogels, a known number of hBMSCs was pelleted and resuspended in the pre-warmed (37 °C) thrombin solution. The pre-warmed fibrinogen solution was then added at a 1:1 ratio and briefly mixed to obtain a final gel concentration of 25 mg/mL. For the final concentration of 50 mg/mL a 100 mg/mL fibrinogen stock solution was used. The fibrinogen–thrombin–cell suspension was then cast into silicone molds to form hydrogels for subsequent culture and mechanical loading. Unless otherwise specified, the fibrinogen–thrombin–cell suspension was rapidly cast (within 3–4 min) into silicone molds (60 µL/well; Ø = 5 mm, h = 3 mm). Gels were allowed to polymerize for 40 min at 37 °C in a humidified incubator.

All constructs were equilibrated in chondrogenic differentiation medium without TGF-β3 (CDM-), consisting of high-glucose DMEM supplemented with 100 U/mL penicillin, 100 µg/mL streptomycin (both Gibco), 100 µg/mL sodium pyruvate, 40 µg/mL L-proline, 50 µg/mL L-ascorbic acid-2-phosphate, 4.7 µg/mL linoleic acid, 1.5 µg/mL BSA, 1× insulin–transferrin–selenium (Thermo Fisher, Waltham, MA, USA), and 100 nM dexamethasone (all Sigma-Aldrich). For experiments investigating the effects of dynamic compression (DC) in the presence of growth factors, CDM– was supplemented with 10 ng/mL TGF-β3 (CDM+; PeproTech). Each hydrogel was cultured in 2 mL of CDM+ before and during DC application.

For studies assessing the effect of growth factor withdrawal, TGF-β3 was removed from the medium after the designated priming period. In all conditions, medium was exchanged twice per week, and the total volume was normalized to the number of hydrogels per condition. Throughout the experiment, all cells and hydrogels were cultured under physioxia (5% O_2_).

### 2.3. Application of Dynamic Compression (DC)

Dynamic compressive loading was applied using a custom-built bioreactor system placed inside a standard cell culture incubator (37 °C, 95% humidity, 5% CO_2_, 5% O_2_). Unconfined, sinusoidal compression parameters were controlled via a LabVIEW-based graphical user interface developed in-house. Samples were subjected to loading for 2 h per day, either continuously or intermittently, at 10% or 20% strain with a 2% static pre-strain. In the intermittent dynamic compression (IDC) mode, 10 min loading periods alternated with 10 min rest intervals; total active loading time remained 2 h per day. For each condition, free-swelling hydrogels cultured in parallel were included as controls (CTL).

### 2.4. Mechanical Testing

Unconfined compressive testing was performed by placing fibrin hydrogels between two stainless steel platens submerged in phosphate-buffered saline (PBS) at room temperature (RT), using a mechanical testing machine (Zwick Roell, Ulm, Germany). A 10 N load cell was used to determine the Young’s modulus, equilibrium modulus, and dynamic modulus. Unless otherwise specified, the Young’s modulus was calculated by applying a compressive strain of 20% and using the linear region of the stress–strain curve between 10% and 20% strain.

To determine the equilibrium modulus, a stress-relaxation test was performed by compressing the hydrogel to 20% strain and allowing the construct to reach mechanical equilibrium. For dynamic modulus measurements, hydrogels were subjected to five compression cycles at 1% strain and 1 Hz. All tests were performed sequentially, and a pre-load of 0.01 N was applied to ensure full contact between the platens and the hydrogel surface.

### 2.5. Gene Expression Analysis

Total RNA was extracted using TRIzol™ Reagent (Thermo Fisher) according to the manufacturer’s instructions. Prior to extraction, samples were physically disrupted using sterile pestles to ensure uniform cell and tissue lysis. After elution and quantification via NanoDrop, equal amounts of RNA from each sample were reverse transcribed into complementary DNA (cDNA) using the High-Capacity cDNA Reverse Transcription Kit (Applied Biosystems™, Carlsbad, CA, USA). The resulting cDNA was quantified using the Qubit™ ssDNA Assay Kit (Thermo Fisher), and 10 ng of cDNA per sample was used for real-time PCR. List of primers can be found in Table 1.

Amplification was carried out using SYBR™ Select Master Mix on a 7500 Fast Real-Time PCR System (Applied Biosystems™). Ribosomal protein L4 (*RPL4*) was used as the housekeeping gene in experiments without TGF-β3; β2-Microglobulin (*B2M*) was used in experiments with TGF-β3. The relative gene expression was calculated using the 2^−ΔΔCt^ method, if not mentioned otherwise.

### 2.6. Quantitative Biochemical Analysis

Samples were halved and digested overnight at 60 °C in a digestion buffer containing 50 mM Na_2_HPO_4_, 50 mM NaH_2_PO_4_, 5 mM EDTA, 10 mM L-cysteine, and 3.88 U/mL papain (pH 6.4; all Sigma-Aldrich). DNA content was assessed using the Hoechst 33258-based quantification kit according to the manufacturer’s instructions (Sigma-Aldrich). Sulfated glycosaminoglycan (sGAG) content was determined using the 1,9-dimethylmethylene blue (DMMB) assay at pH 1.5; metachromatic shifts were quantified spectrophotometrically at 530 and 590 nm. Shark chondroitin sulfate (Sigma-Aldrich) was used to generate the standard curve.

For collagen quantification, digested samples were hydrolyzed overnight in 38% HCl, then oxidized with 41 mM chloramine-T to form pyrrole-2-carboxylate. Indole derivatives were subsequently quantified using Ehrlich’s reagent, which contained 2 M 4-dimethylaminobenzaldehyde, with absorbance measured at 570 nm. Trans-4-hydroxy-L-proline (Sigma-Aldrich) was used as the standard, and collagen content was calculated using a hydroxyproline-to-collagen ratio of 1:7.69 [36]. All spectrophotometric measurements were performed using a Synergy HT multi-detection plate reader (BioTek Instruments, Inc., Winooski, VT, USA).

### 2.7. Histological Analysis

Halved samples were fixed in 4% paraformaldehyde overnight at 4 °C. Following fixation, samples were dehydrated through a graded ethanol series (50–100%), cleared with xylene, and infiltrated with paraffin wax. Dehydration, clearing, and infiltration steps were performed using an automated tissue processor (Leica Biosystems, Nussloch, Germany). Samples were then embedded in paraffin blocks and sectioned at 5 µm using a microtome (Leica Biosystems).

All histological stains were applied using an autostainer (Leica Biosystems). To assess sulfated glycosaminoglycan (sGAG) content, sections were stained with Alcian Blue (AB) at pH 1.0 (1% *w*/*v* Alcian Blue 8GX in 0.1 M HCl), and counterstained with Nuclear Fast Red (0.1% *w*/*v*). Collagen deposition was visualized using Picrosirius Red (PR; 0.1% *w*/*v*), and calcium deposition was assessed using Alizarin Red (AR; 1% *w*/*v*, pH 4.1). All staining reagents were obtained from Sigma-Aldrich. Stained slides were imaged using an Aperio ScanScope digital slide scanner (Leica Biosystems).

### 2.8. Immunohistochemistry

After deparaffinization and rehydration, antigen retrieval was performed by sequential incubation with 1% (*w*/*v*) hyaluronidase (Sigma-Aldrich) and 3.5 U/mL pronase (Merck) for 25 min at 37 °C. Sections were then blocked at room temperature (RT) using a solution containing 10% (*v*/*v*) goat serum and 1% (*w*/*v*) bovine serum albumin (BSA; Sigma-Aldrich), and subsequently incubated overnight at 4 °C with mouse primary antibodies diluted in the same blocking buffer.

The following primary antibodies were used: anti-collagen type II (Col2; SC-52658, 1:200), anti-collagen type I (Col1; ab138492, 1:200), and anti-collagen type X (Col10; ab49945, 1:200). For detection, the following secondary antibodies were applied for 4 h at RT: goat anti-mouse IgG (A11001) for Col2, goat anti-mouse IgM (ab150121) for Col10, and donkey anti-rabbit IgG (ab150075) for Col1. Nuclei were counterstained with DAPI (2 µg/mL; Sigma-Aldrich), and slides were mounted using ProLong™ Gold Antifade Mountant (Invitrogen, Carlsbad, CA, USA).

Immunostained samples were imaged using a Leica SP8 scanning confocal microscope. Unless otherwise specified, images are shown as maximum intensity projections of comparable Z-stacks and were processed using Fiji (ImageJ, version 1.54f, National Institutes of Health, Bethesda, MD, USA).

### 2.9. Statistical Analysis

Statistical analysis was performed using GraphPad Prism (GraphPad Software, version 8.4.3, Boston, MA, USA). All experiments were conducted with at least three technical replicates per condition. Comparisons between two groups were evaluated using unpaired two-tailed Student’s *t*-tests. For comparisons among multiple groups, one-way analysis of variance (ANOVA) followed by Tukey’s post hoc multiple comparison test was used. Two-way ANOVA with Tukey’s post hoc test was applied when analyzing the effects of two independent variables (e.g., stimulation and duration). Data are presented as mean ± standard deviation (SD), and differences were considered statistically significant at *p* ≤ 0.05.

## 3. Results

### 3.1. Dynamic Compression Alone Does Not Initiate Chondrogenic Differentiation of hMSCs

**Figure 1:**
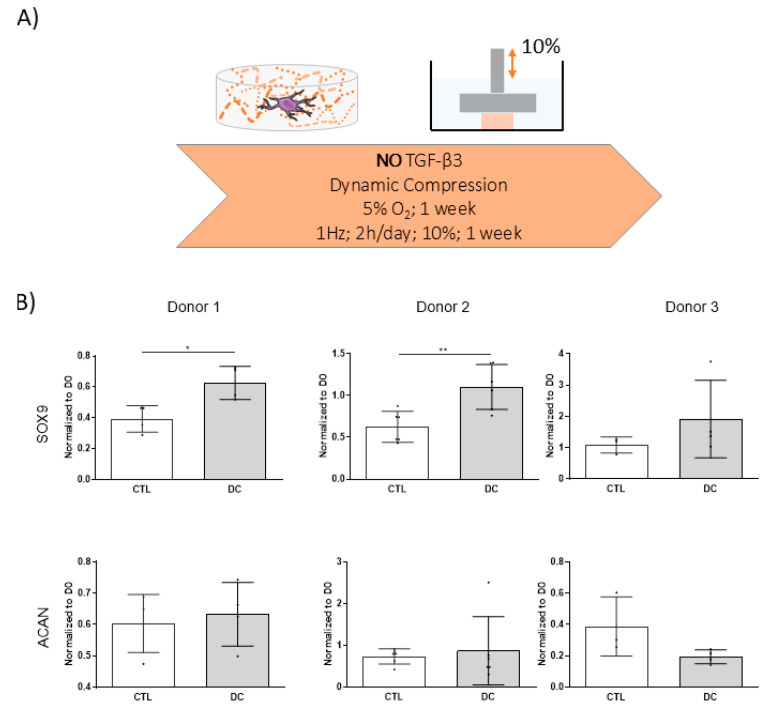
Dynamic compression does not initiate hMSC chondrogenesis in absence of TGF-β3. (**A**) Experimental setup. hMSCs were encapsulated in 5% fibrin hydrogels at a density of 15 × 10^6^ cells/mL and subjected to dynamic compression (1 Hz, 2 h/day) for 5 days in chemically defined media without TGF-β3. (**B**) Relative gene expression levels of chondrogenic markers (*SOX9*, *ACAN*) were determined using the 2^−ΔΔCt^ method and normalized to day 0. *RPL4* functioned as the housekeeping gene. Corresponding 2^−ΔCt^ values are provided in Supplementary Figure S1. All data represented as mean ± SD; *n* ≥ 3; * *p* ≤ 0.05; ** *p* ≤ 0.01.

DC has been shown to support in vitro chondrogenesis, yet it remains unclear whether such cues alone are sufficient to initiate differentiation in the absence of soluble growth factors. To assess whether DC alone is sufficient to initiate chondrogenesis, hMSCs were encapsulated at a density of 15 × 10^6^ cells/mL in 5% fibrin hydrogels and subjected to DC loading in the absence of TGF-β3 (Figure 1A). Gene expression analysis revealed that *SOX9* was significantly upregulated in 2 out of 3 evaluated donors following the application of DC (Figure 1B). However, this upregulation was modest and remained comparable to or below expression levels on day 0. Furthermore, no significant changes in *ACAN* expression were observed, and *COL2A1* expression was consistently below the detection limit across all samples. These findings indicate that, in this experimental framework, DC alone is insufficient to initiate chondrogenesis. However, it should be noted that using only three donors provides limited statistical power, and larger donor numbers will be required for more conclusive interpretation.

### 3.2. Higher Seeding Densities Support Superior TGF-β3-Mediated Chondrogenesis

**Figure 2:**
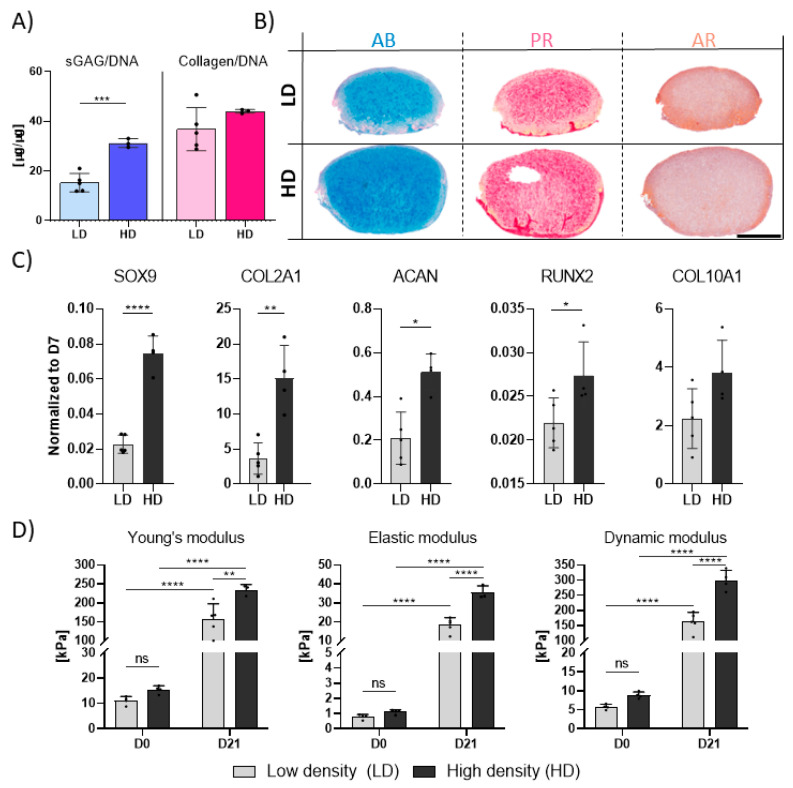
Chondrogenic differentiation of hMSCs at low (LD: 4.17 × 10^6^ cells/mL) and high (HD: 8.33 × 10^6^ cells/mL) densities over 3 weeks. (**A**) Biochemical content analysis; sGAG levels were determined via DMMB assay; collagen levels were determined by quantifying hydroxyproline levels. Values were normalized to the DNA content. (**B**) Histological assessment of ECM components. AB: Alcian blue; PR: Picrosirius red; AR: Alizarin red. (**C**) Gene expression analysis of chondrogenic and hypertrophic markers. Relative gene expression levels were determined by using the 2^−ΔCt^ method. Housekeeping gene: *B2M*. (**D**) Assessment of mechanical properties; Young’s, equilibrium and dynamic moduli were determined in an unconfined compression configuration. All data represented as mean ± SD; *n* ≥ 3; * *p* ≤ 0.05; ** *p* ≤ 0.01; *** *p* ≤ 0.001; **** *p* ≤ 0.0001; ns: not significant Scale bar = 1 mm.

Altering cell density is perhaps one of the simplest strategies to steer cartilage graft development. While hydrogel seeding density has been shown to affect MSC chondrogenesis, the mechanoresponsiveness of such engineered grafts remains poorly understood. To examine this interplay, we first sought to confirm the differential chondrogenic capacity of MSCs encapsulated into fibrin hydrogels at two seeding densities in static conditions. MSCs were encapsulated in 2.5% fibrin hydrogels at either 4.17 × 10^6^ cells/mL (Low Density: LD) or 8.33 × 10^6^ cells/mL (High Density: HD) and cultured statically in chondrogenic media supplemented with TGF-β3 for three weeks. Higher density constructs exhibited significantly enhanced chondrogenic differentiation compared to lower density samples (Figure 2). Although no differences in overall collagen deposition were observed, sGAG deposition (when normalized to DNA content) was significantly higher in HD constructs (Figure 2 A,B). Gene expression analysis further supported this observation, with *SOX9*, *ACAN* and *COL2A1* significantly upregulated in the HD condition (Figure 2C). In line with these findings, Young’s and dynamic moduli of HD samples were significantly higher than of LD samples (Figure 2D).

### 3.3. Higher Magnitudes of DC Are Not Beneficial to TGF-β3-Mediated Chondrogenesis

**Figure 3:**
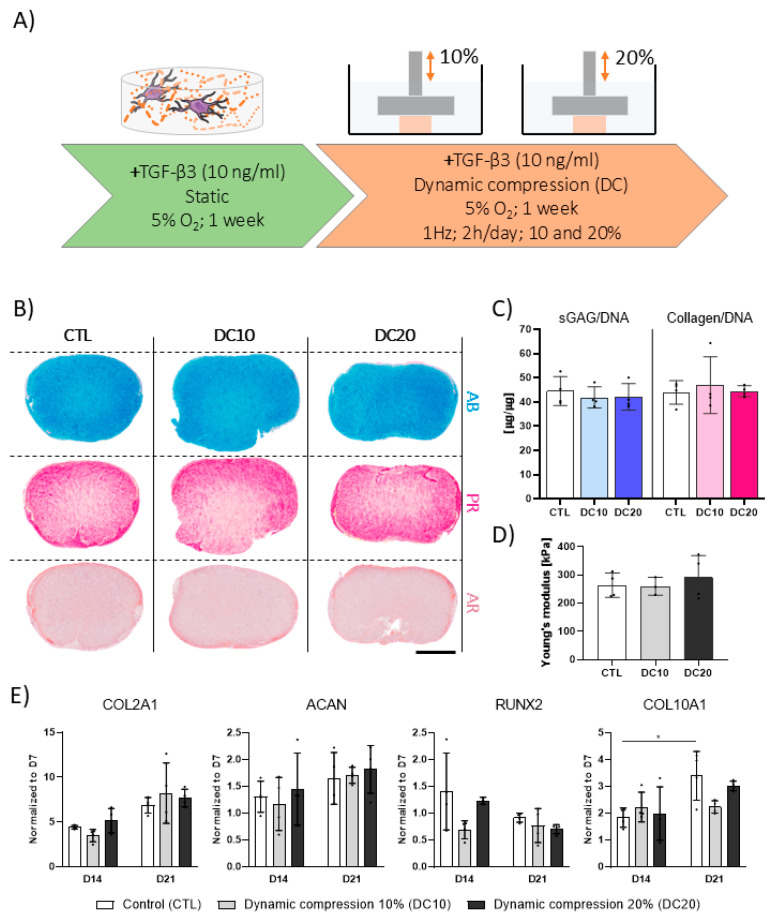
The effect of DC on TGF-β3 mediated chondrogenesis of hMSCs. (**A**) Human MSCs were encapsulated in 2.5% fibrin hydrogels (HD: 8.33 × 10^6^ cells/mL) and subjected to DC (10% and 20% strain) at 1 Hz for 2 h per day, following a one-week priming period. TGF-β3 (10 ng/mL) was supplemented at throughout the duration of the experiment. (**B**) Histological analysis of ECM components. AB: Alcian blue; PR: Picrosirius red; AR: Alizarin red. (**C**) Biochemical content analysis. sGAG levels were determined via DMMB assay; collagen levels were determined by quantifying hydroxyproline levels. Values were normalized to DNA content. (**D**) Young’s modulus of cartilage grafts after three weeks in culture. The stiffness was determined using the linear region of the stress–strain curve between 10% and 20%. CTL: Control. DC10: 10%. DC20: 20%. (**E**) Relative gene expression levels of chondrogenic (*COL2A1*, *ACAN*) and hypertrophic (*RUNX2*, *COL10A1*) markers. Expression was determined using the 2^−ΔΔCt^ method and normalized to day 7, with *B2M* as the housekeeping gene. Corresponding 2^−ΔCt^ values are provided in Supplementary Figure S2. All data represented as mean ± SD; *n* ≥ 3; * *p* ≤ 0.05. Scale bar = 1 mm.

Having demonstrated superior chondrogenesis of hMSCs at high seeding densities, we next examined whether the application of different DC magnitudes affects chondrogenic differentiation. To this end, high-density samples were subjected to 10% or 20% strain DC for two weeks following an initial one-week priming period in static conditions (Figure 3A). Although robust chondrogenesis was observed for all conditions based on histological and biochemical analyses, the application of DC at either 10% or 20% strain did not improve the deposition of sGAG or collagens. Similarly, DC did not improve graft mechanical properties (Figure 3B–D). These results were further confirmed by gene expression analysis; DC did not influence the expression of chondrogenic genes at any of the examined timepoints (Figure 3E).

### 3.4. DC Regulates TGF-β3 Mediated Chondrogenesis of hMSCs in a Cell Density-Dependent Manner

**Figure 4:**
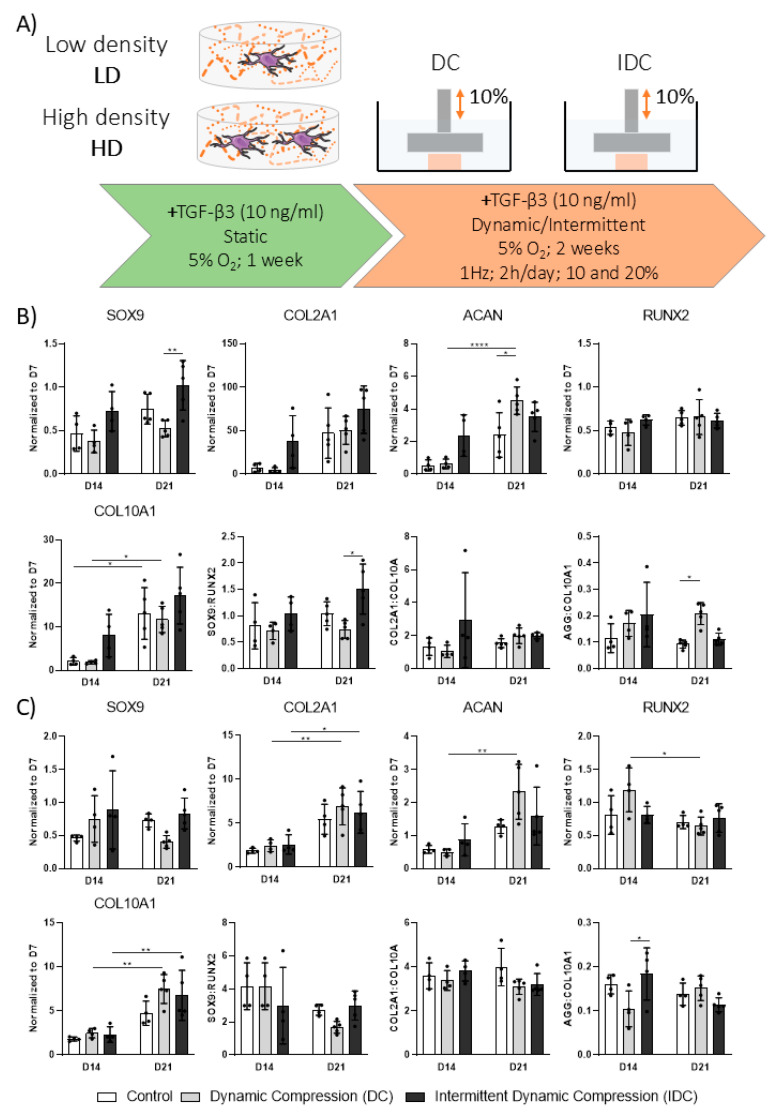
(**A**) Human MSCs were encapsulated in 2.5% fibrin hydrogels at two cell densities (low density, LD: 4.17 × 10^6^ cells/mL and high density, HD: 8.33 × 10^6^ cells/mL) and were subjected to either continuous Dynamic Compression (DC) or Intermittent Dynamic Compression (IDC) at 1Hz. IDC loading regime consisted of alternating 10 min compression periods and 10 min break intervals. Total loading time was identical for both DC and IDC: 2 h/day. TGF-β3 was supplemented at 10 ng/mL throughout the three-week duration of the experiment. (**B**) Gene expression analysis: LD. (**C**) Gene expression analysis: HD. Relative gene expression levels of chondrogenic (*SOX9*, *ACAN*, *COL2A1*) and hypertrophic (*RUNX2*, *COL10A1*) markers were determined using the 2^−ΔΔCt^ method. All values were normalized to day 7, with *B2M* as the housekeeping gene. Corresponding 2^−ΔCt^ values are provided in Supplementary Figure S3. All data represented as mean ± SD; *n* = 4; * *p* ≤ 0.05, ** *p* ≤ 0.01, **** *p* ≤ 0.0001.

Given the effect of cell density on chondrogenic differentiation in static conditions, we next sought to investigate whether this variable also influences the mechanoresponsiveness of engineered constructs. To this end, hMSCs were encapsulated in 2.5% fibrin hydrogels at previously established cell densities and were subjected to 10% strain DC following a one-week priming period in chondrogenic medium (Figure 4A). Given the viscoelastic nature of fibrin, which we observed to deform progressively during sustained loading, an intermittent dynamic compression (IDC) regime was also introduced. This approach incorporated short recovery intervals intended to allow hydrogel recovery and potentially enhance mechanotransduction.

The expression of key chondrogenic and hypertrophic genes were not affected by either continuous DC or IDC on day 21 at higher densities (Figure 4B,C). However, at lower densities, DC resulted in a moderate yet significant increase in *ACAN* expression by day 21. Although *SOX9* was significantly upregulated in low-density constructs subjected to IDC on day 21 compared to DC constructs, the levels were comparable to day 7.

**Figure 5:**
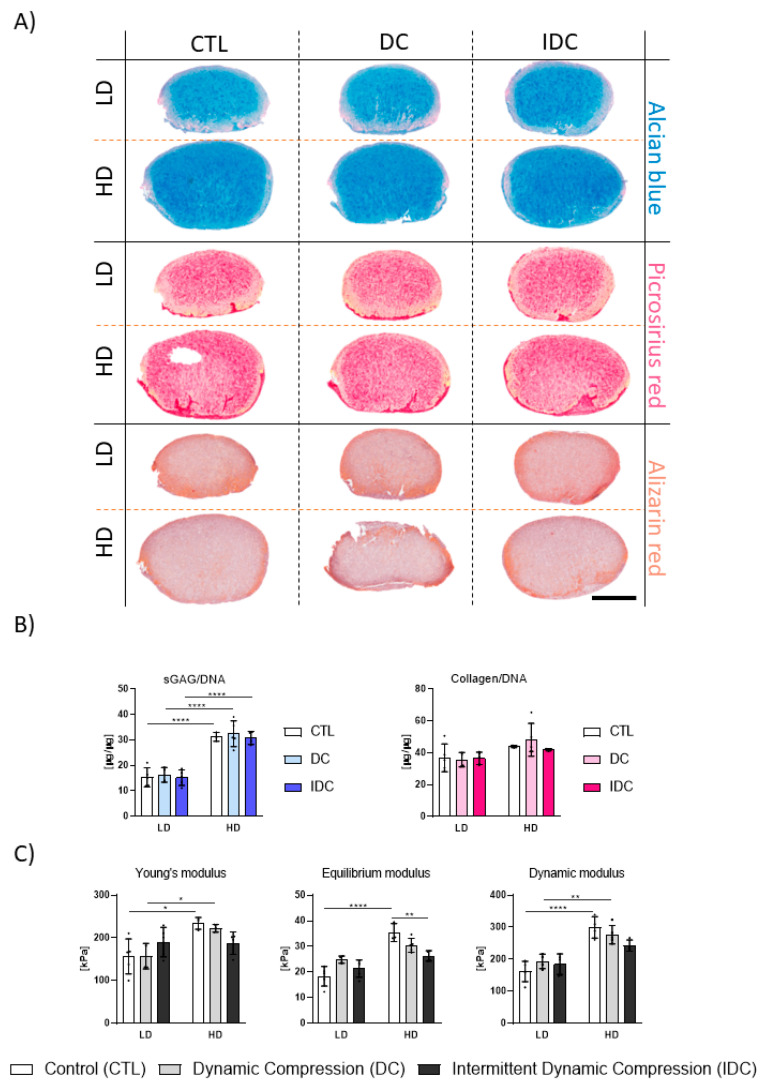
Influence of DC and IDC on deposition of ECM at different cell densities after one week of chondrogenic priming. (**A**) Histological analysis. AB: Alcian blue; PR: Picrosirius red; AR: Alizarin red. Scale bar = 1 mm. (**B**) Biochemical content analysis after 21 days in culture. sGAG levels were determined using DMMB assay; collagen levels were quantified via hydroxyproline measurement. (**C**) Assessment of mechanical properties: Young’s, equilibrium, and dynamic moduli in unconfined compression of hMSCs encapsulated in 2.5% fibrin hydrogels. Low density (LD): 4.17 × 10^6^ cells/mL. High density (HD): 8.33 × 10^6^ cells/mL. CTL: Control. DC: Dynamic compression. IDC: Intermittent dynamic compression. All data represented as mean ± SD; *n* ≥ 3; * *p* ≤ 0.05; ** *p* ≤ 0.01; **** *p* ≤ 0.0001.

An analysis of key gene ratios did not reveal any notable trends towards either chondrogenic or hypertrophic phenotypes when subjected to DC. However, the *SOX9*/*RUNX2* ratio appeared to be positively affected by the intermittent compression in low-density samples on day 21 (Figure 4B,C). Furthermore, the ratios of *SOX9*/*RUNX2* and *COL2A1*/*COL10A1* in the high-density groups indicate an overall more chondrogenic state compared to low-density samples. Robust chondrogenic differentiation was also confirmed by a biochemical analysis of ECM deposition within the fibrin hydrogels (Figure 5A,B), although the addition of either continuous or intermittent DC did not significantly affect the deposition of sGAG and collagen. Furthermore, no significant changes in mechanical properties were observed in low-density constructs due to the application of mechanical loading (Figure 5C). A significant reduction in the equilibrium modulus of the high-density constructs was observed following the application of IDC.

### 3.5. DC Can Be Detrimental to hMSC Chondrogenesis upon Removal of Exogenous TGF-β3

**Figure 6:**
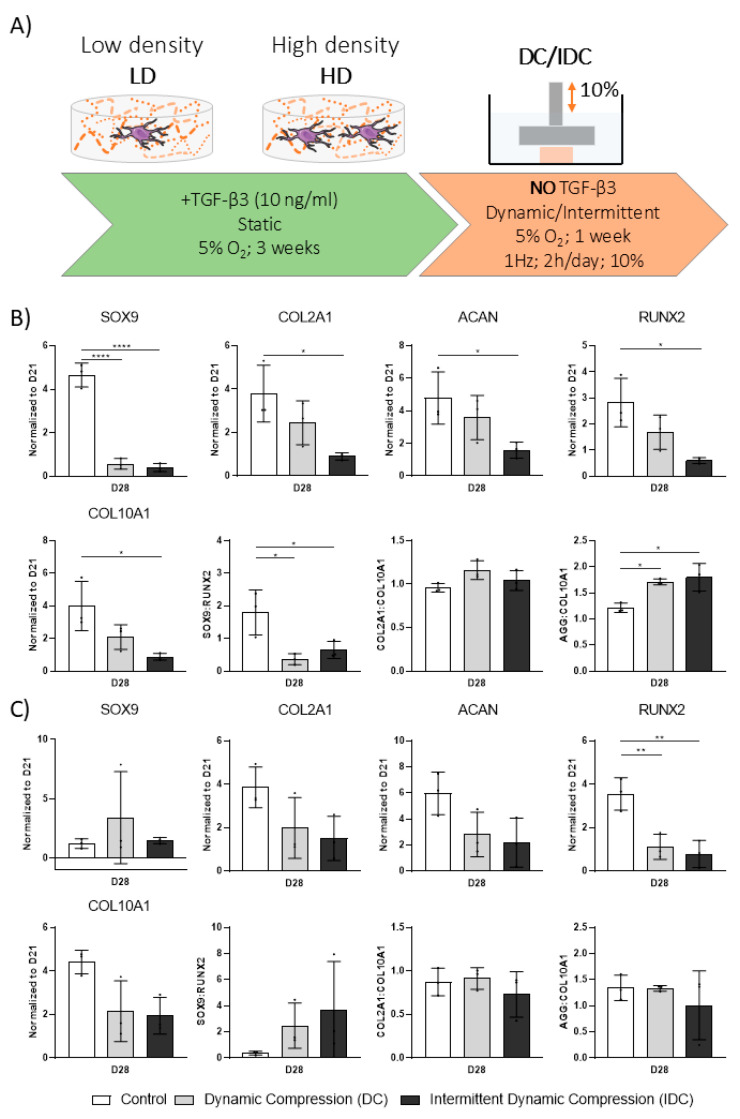
(**A**) Human MSCs were encapsulated in 2.5% fibrin hydrogels at two cell densities and were cultured for three weeks in presence of TGF-β3. In the fourth week, TGF-β3 was withdrawn and mechanical compression was applied. Continuous Dynamic Compression (DC) or Intermittent Dynamic Compression (IDC) were applied at 1 Hz. IDC loading regime consisted of alternating 10 min compression and 10 min break intervals. Total loading time for both conditions was identical: 2 h/day. Low density (LD): 4.17 × 10^6^ cells/mL. High density (HD): 8.33 × 10^6^ cells/mL. (**B**) Gene expression analysis: LD. (**C**) Gene expression analysis: HD. Relative gene expression levels of chondrogenic (*SOX9*, *ACAN*, *COL2A1*) and hypertrophic (*RUNX2*, *COL10A1*) were determined using 2^−ΔΔCt^ method. Gene expression ratios were determined using 2^−ΔCt^ values. All values were normalized to day 21 with *B2M* as a housekeeping gene. Corresponding 2^−ΔCt^ values are provided in Supplementary Figure S4. All data represented as mean ± SD; *n* = 4; * *p* ≤ 0.05; ** *p* ≤ 0.01; **** *p* ≤ 0.0001.

We next investigated how the removal of growth factors after a priming period of three weeks would affect the response of engineered cartilage grafts to mechanical stimulation. By employing two cell densities and two DC modes, continuous and intermittent, we also aimed to investigate the contribution of these factors to either a chondrogenic or hypertrophic phenotype. For this purpose, hMSCs were encapsulated in 2.5% fibrin hydrogels at two cell densities and were cultured for three weeks in chondrogenic media. Subsequently, these matured grafts were transferred to a bioreactor for dynamic compression in the absence of exogenous TGF-β3 (Figure 6A).

Most chondrogenic and hypertrophic genes were upregulated onday 28 following growth factor withdrawal, with the exception of *SOX9* in high-density cultures (Figure 6B,C). Superimposing mechanical stimulation after the priming period had a substantial impact on both chondrogenic and hypertrophic markers: DC and IDC dramatically suppressed the expression of *SOX9* in the low-density constructs, but not in the high-density groups. This suggests a role of cell density and/or associated construct maturation in determining hMSC mechanoresponsiveness after TGF-β3 priming. Gene expression ratios further confirmed this observation (Figure 6B,C). A larger decrease in the key *SOX9*/*RUNX2* ratio was observed in the low-density samples in response to mechanical stimulation compared to high-density cultures.

**Figure 7:**
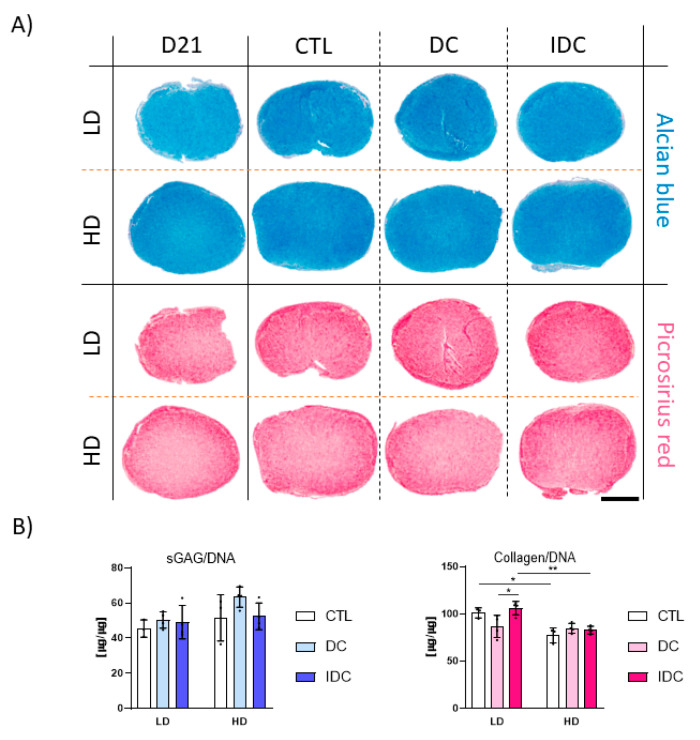
Effect of priming and subsequent mechanical stimulation on deposition of ECM. Analysis after 21 days of priming in chondrogenic media with subsequent 7-day mechanical stimulation in absence of TGF-β3. (**A**) Histological analysis; AB: Alcian blue; PR: Picrosirius red. (**B**) Biochemical content analysis; DNA levels were determined using Hoechst-based assay. sGAG levels were determined using DMMB assay. Collagen levels were determined by quantifying hydroxyproline content. Low density (LD): 4.17 × 10^6^ cells/mL. High density (HD): 8.33 × 10^6^ cells/mL. All data represented as mean ± SD; *n* ≥ 3; * *p* ≤ 0.05; ** *p* ≤ 0.01. Scale bar = 1 mm.

**Figure 8:**
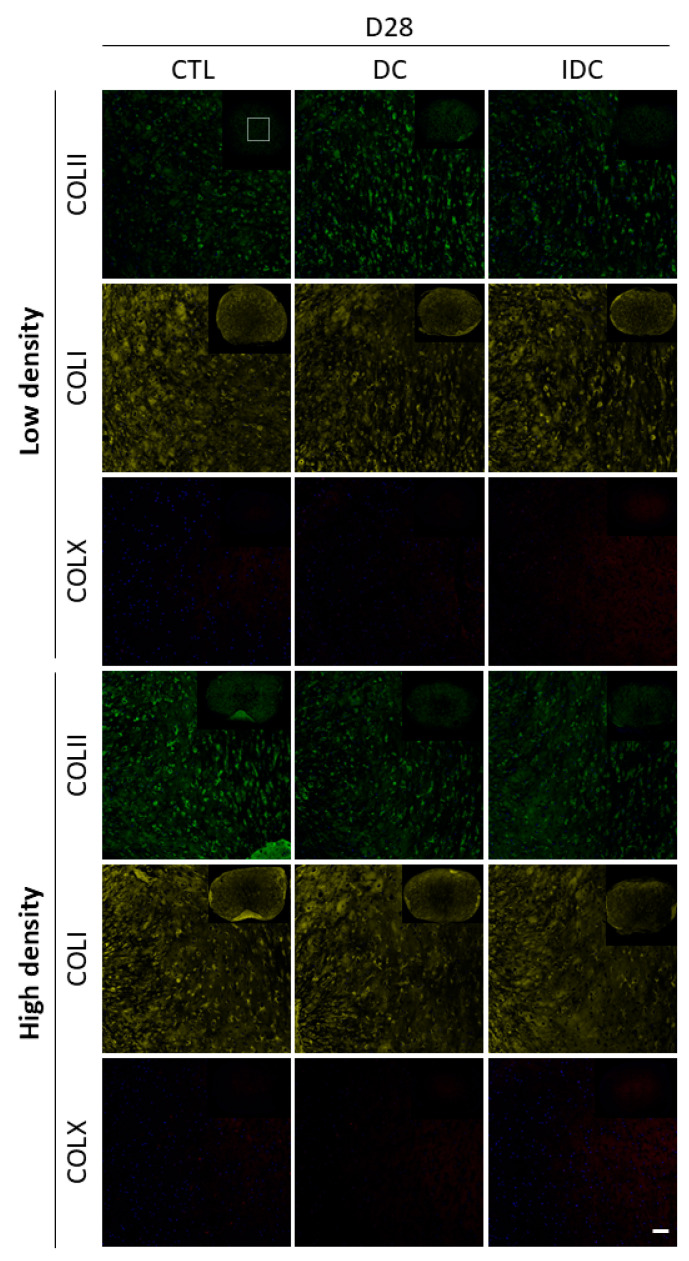
Immunofluorescence analysis of collagen subtype deposition. Human MSCs were subjected to mechanical stimulation after a three-week priming period with TGF-β3. Confocal images for collagen type II (green), collagen type I (yellow) and collagen type X (red). CTL: Control. DC: Dynamic compression. IDC: Intermittent dynamic compression. Low density (LD): 4.17 × 10^6^ cells/mL. High density (HD): 8.33 × 10^6^ cells/mL. Scale bar = 100 µm.

DC or IDC did not appear to influence the bulk biochemical composition of engineered grafts after the growth factor priming period, as evidenced by biochemical and immunohistological analyses (Figure 7). No clear changes in the deposition of different collagen subtypes were observed in response to mechanical stimulation (Figure 8). Signal intensities for collagen types I, II and X were comparable across control (CTL), DC and IDC conditions on day 28. Notably, collagen type X levels remained low under all conditions. Lastly, changes in cell density did not visibly affect the deposition of collagen types I or X; both were present at similar levels. However, collagen type II staining was more intense in high-density samples, with a less punctate, more diffuse, and homogeneous distribution throughout the tissue.

## 4. Discussion

DC is one of the key mechanical forces experienced by articular cartilage during daily joint loading. Bioreactors have been developed to recapitulate aspects of such physiological cues, which can be used to study the influence of DC on the chondrogenic differentiation of MSCs in the context of articular cartilage tissue engineering [26,37]. Although mechanical stimulation has been shown to be pivotal during development and regeneration in vivo, the benefits of mechanical compression for in vitro hMSC chondrogenesis remain unclear [14,15]. Therefore, this study examined whether DC alone could initiate chondrogenesis and how continuous and intermittent DC interacts with TGF-β3 to regulate chondrogenesis in 3D. We also explored whether the withdrawal of TGF-β3 after a priming period would affect the mechanoresponsiveness of engineered cartilage grafts, thereby partially mimicking the transition from controlled in vitro culture to the mechanically demanding in vivo environment.

The initial experiment in this study aimed to determine whether DC alone could initiate chondrogenic differentiation of hMSCs. Although DC led to a modest upregulation of *SOX9* in some donors, no induction of matrix-associated markers such as *ACAN* or *COL2A1* was observed. These findings are consistent with a recent study using hydrostatic pressure, where mechanical stimulation alone induced *SOX9* expression but failed to induce robust chondrogenesis in the absence of TGF-β3 [38]. While additional studies are needed to assess the influence of parameters such as cell density, material stiffness, and loading profile, the data from three donors indicated that DC alone is insufficient to initiate chondrogenesis. This agrees with previous studies which have found that the application of both compression and shear is required to initiate chondrogenesis in the absence of exogenously supplied growth factors [39].

Cell density is one of the simplest fundamental culture parameters that can be adjusted to engineer constructs with distinct biochemical compositions. There appears to be an intricate relationship between cell density and ECM output, where higher cell numbers correlate positively with sGAG but not collagen deposition [6]. Increasing the cell density will increase the overall nutrient demand of the construct, reducing the levels of oxygen and other regulatory factors within the developing tissue, conditions which may preferentially support sGAG over collagen deposition [40]. Given the importance of ECM composition for mechanoresponsiveness, we further explored how these density-dependent differences interact with dynamic compression.

The application of DC, either continuous or intermittent, during TGF-β3-mediated chondrogenic differentiation did not result in significant changes in ECM synthesis in either low- or high- cell density conditions. Furthermore, following the removal of TGF-β3 after a priming period, the application of DC did not markedly alter overall ECM deposition at either cell density. However, gene expression analysis revealed a general downregulation of chondrogenic and hypertrophic markers in response to mechanical stimulation, particularly in the less mature tissues formed under low cell-density conditions. This is also reflected in marker ratios (*SOX9*/*RUNX2*, *COL2A1*/*COL10A1),* which pointed to a more chondrogenic state at high density, while low-density constructs were more affected by loading.

A number of previous studies have reported that applying DC enhances TGF-β3 mediated chondrogenic differentiation of MSCs [26,37,41,42,43]. The applied DC strain in this study represents the commonly used compression range of 10–20%, which enables comparison across studies while remaining physiologically relevant [26,44]. However, in the present study, subjecting hMSCs encapsulated in fibrin to 10% or 20% strain DC did not improve ECM deposition or the resulting mechanical properties of the engineered graft. Although experimental concepts applied in the literature are often comparable, distinct differences regarding scaffold type, cell density, duration of loading, and cell source are likely contributors to the observed discrepancies [45]. These variations highlight the challenge of identifying universal parameters for promoting mechanoresponsiveness in vitro and emphasize the need for greater standardization in mechanical stimulation studies [27].

The majority of studies examining the effects of mechanical compression have focused on how DC influences chondrogenesis when applied in the presence of growth factors [13,29,46]. However, relatively little is known about how DC influences chondrogenesis once TGF-β3 is withdrawn following the induction of differentiation [47]. This question is particularly relevant for translational applications, as many tissue engineering strategies involving hMSCs will likely require in vitro maturation of grafts, followed by implantation into mechanically demanding environments without exogenous TGF-β3 supplementation. In this context, DC bioreactors offer a valuable platform to partially simulate this transition and to inform improved in vitro conditioning protocols for articular cartilage tissue engineering. The removal of growth factors after 21 days of priming did not result in a decrease in chondrogenic or hypertrophic gene expression by day 28 in static conditions. On the contrary, relative levels of *COL2A1*, *ACAN*, *RUNX2*, and *COL10A1* increased in both low- and high-density cultures. This suggests that continuous supplementation with TGF-β3 may not be necessary, and that a pulsed or transient exposure could be sufficient to induce robust chondrogenesis under certain conditions [38,48]. More importantly, the application of mechanical stimulation in the absence of TGF-β3 appeared to negatively affect gene expression, particularly in low-density constructs. *SOX9* expression was most affected in these grafts, indicating they may be least suitable for in vivo implantation. This effect could be linked to lower levels of pericellular and extracellular matrix components in low-density samples. Given the protective role of sGAGs against compressive forces, it is feasible that insufficient matrix deposition at this stage rendered low-density constructs more susceptible, resulting in a catabolic effect [49]. This effect may have been further amplified by the reduced autocrine/paracrine activity of low-density cultures, with lower availability of secreted factors under DC [50]. These findings are consistent with our previous work, where hydrostatic pressure proved more beneficial at later stages of maturation [38]. Although these changes were not evident in histological or biochemical analyses, it is important to note that grafts were exposed to only one week of mechanical loading; whereby longer stimulation periods may reveal differences at the protein level [13]. In this context, more targeted quantification of proteins in future studies (e.g., Western blotting) would provide additional insights into the DC impact.

These findings support the hypothesis that low-density cultures represent suboptimal conditions for hMSC chondrogenesis compared to high-density cultures, which appeared more mature based on sGAG and collagen type II accumulation at the end of the culture period. A comparable study using synovium-derived stem cells (SMSCs) in agarose hydrogels demonstrated that DC applied after 21 days of priming elicited greater expression of chondrogenic markers than DC applied at the onset of differentiation [51]. While differences in cell type, biomaterial, and loading parameters must be considered, this maturity-dependent mechanoresponsiveness underscores the critical role of priming time and construct maturation in determining the effectiveness of mechanical loading. We observed similar trends in a separate study using a hydrostatic pressure bioreactor, where more mature cartilage grafts also exhibited enhanced responses to mechanical stimulation [6,38].

## 5. Conclusions

This study investigated how fundamental cell culture variables, such as cell density and growth factor availability, influence the mechanoresponsiveness of chondrogenically differentiating hMSCs to (DC). To this end, three articular cartilage tissue engineering relevant scenarios were explored. First, we examined whether DC could initiate chondrogenesis without TGF-β3 supplementation. Despite upregulation of *SOX9*, robust expression of other chondrogenic markers could not be achieved. Second, when DC was applied concurrently with TGF-β3, mechanical stimulation appeared to transiently enhance gene expression in low-density constructs, suggesting a potential benefit under suboptimal conditions. Lastly, we employed a DC bioreactor to simulate the in vivo transition following implantation, characterized by the withdrawal of supraphysiological growth factors and exposure to mechanical loading. Under these conditions, DC negatively affected gene expression in both low- and high-density constructs, with less mature, low-density tissues being the most impacted. These findings suggest that insufficiently matured grafts may be poorly suited for implantation into load-bearing cartilage defects. Together, the results underscore the importance of culture optimization and preconditioning strategies in the development of functional and mechanically robust cartilage grafts.

## Figures and Tables

**Table 1:** List of employed primers

Gene Name	Forward/Reverse
*SOX9*	F: 5′-CTCTGGAGACTTCTGAACG R: 5′-AGATGTGCGTCTGCTC
*RUNX2*	F: 5′-AAGCTTGATGACTCTAAACC R: 5′-TCTGTAATCTGACTCTGTCC
*COL2A1*	F: 5′-GAAGAGTGGAGACTACTG R: 5′-CAGATGTGTTTCTTCTCCTG
*ACAN*	F: 5′-CACCCCATGCAATTTGAG R: 5′-AGATCATCACCACACAGTC
*COL10A1*	F: 5′-GCTAGTATCCTTGAACTTGG R: 5′-CCTTTACTCTTTATGGTGTAGG
*B2M*	F: 5′-AAGGACTGGTCTTTCTATCTC R: 5′-GATCCCACTTAACTATCTTGG
*RPL4*	F: 5′-GTAACTACAATCTTCCCATGC R: 5′-GGTCTTTGCATATGGGTTTAG

## Data Availability

Data will be made available on request.

## Supplementary Materials

The following supporting information can be downloaded at: https://www.mdpi.com/article/10.3390/bioengineering12101075/s1, Figure S1: Dynamic compression does not initiate hMSC chondrogenesis in absence of TGF-β3. Relative gene expression levels of chondrogenic markers were calculated using the 2^-ΔCt^ method; Figure S2: The effect of DC on TGF-β3 mediated chondrogenesis of hMSCs. Relative gene expression levels of chondrogenic and hypertrophic markers were calculated using the 2^-ΔCt^ method; Figure S3: Human MSCs were encapsulated in fibrin hydrogels at two cell densities and were subjected to either continuous Dynamic Compression (DC) or Intermittent Dynamic Compression (IDC) in presence of TGF-β3. Relative gene expression levels of chondrogenic and hypertrophic markers were calculated using the 2^-ΔCt^ method; Figure S4: Human MSCs were encapsulated in fibrin hydrogels at two cell densities and were cultured for three weeks in presence of TGF-β3. In the fourth week, TGF-β3 was withdrawn and mechanical compression was applied; continuous Dynamic Compression (DC) or Intermittent Dynamic Compression (IDC). Relative gene expression levels of chondrogenic and hypertrophic markers were calculated using the 2^-ΔCt^ method.
