# Supplementary material for: Chondrogenic Maturation Governs hMSC Mechanoresponsiveness to Dynamic Compression
Source: Bioengineering (Basel). 2025 Oct 3;12(10):1075. doi: 10.3390/bioengineering12101075 (PMC12561620; doi:10.3390/bioengineering12101075)
Supplement: Supplementary file 1 [file bioengineering-12-01075-s001.zip › bioengineering-3865128-supplementary.pdf]

Supplementary Figures

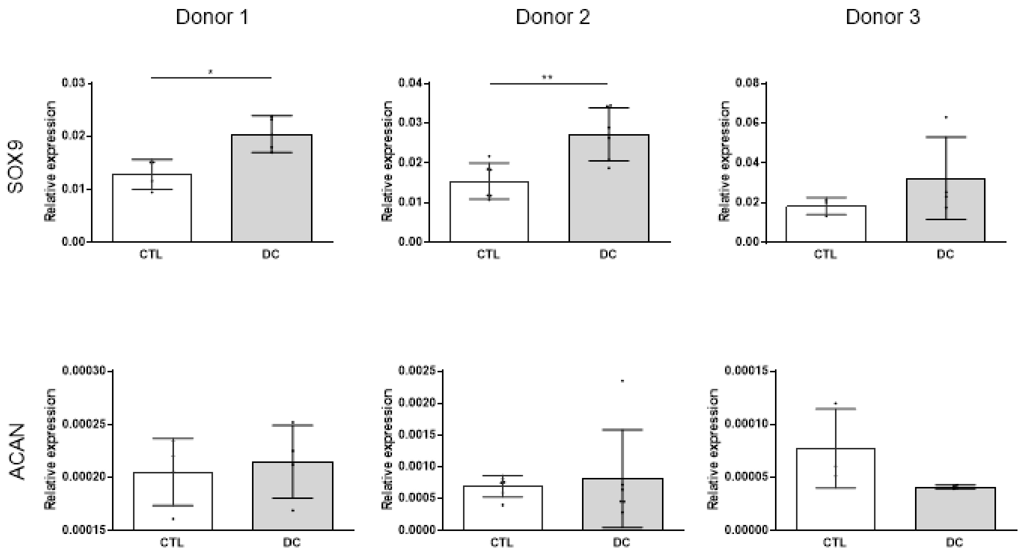

Supplementary figure S1: Dynamic compression does not initiate hMSC chondrogenesis in absence of TGF-β3. Relative gene expression levels of chondrogenic markers (SOX9, ACAN) were determined using the $2^{-\Delta Ct}$ method and normalized to the housekeeping gene RPL4. All data represented as mean ± SD; n≥3; *p≤0.05; **p≤0.01.

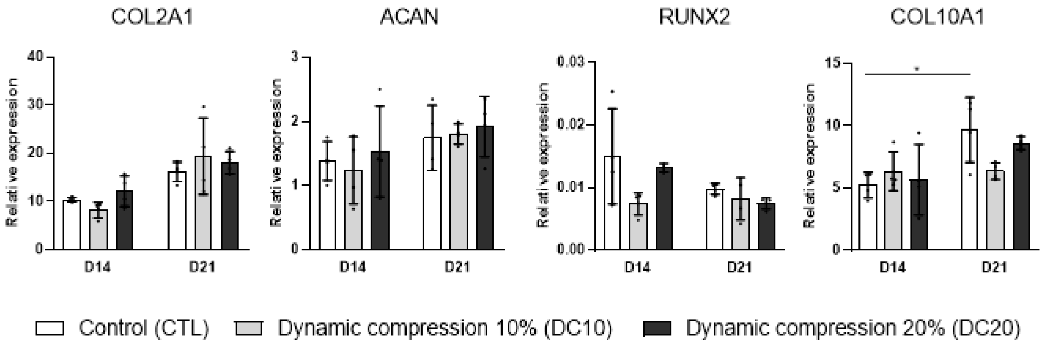

Supplementary figure S2: The effect of DC on TGF-β3 mediated chondrogenesis of hMSCs. Human MSCs were encapsulated in 2.5% fibrin hydrogels (HD: $8.33*10^6$ cells/mL) and subjected to DC (10% and 20% strain) at 1 Hz for 2 hours per day, following a one-week priming period. Relative gene expression levels of chondrogenic (COL2A1, ACAN) and hypertrophic (RUNX2, COL10A1) markers. Expression was determined using the $2^{-\Delta Ct}$ method and normalized to the housekeeping gene B2M. All data represented as mean ± SD; n≥3; *p≤0.05.

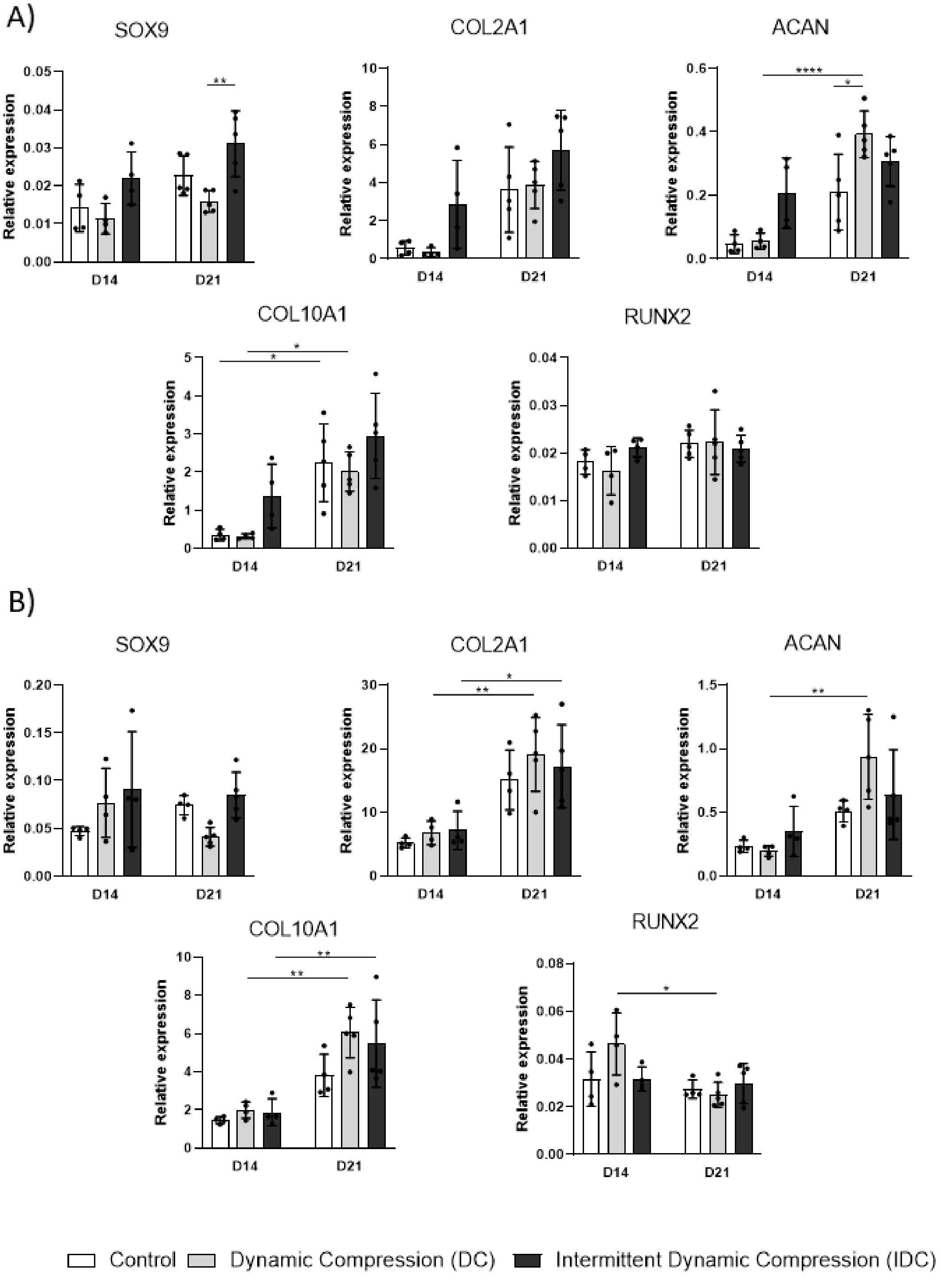

Supplementary figure S3: Human MSCs were encapsulated in 2.5 % fibrin hydrogels at two cell densities (A) LD: 4.17*10^6 cells/mL and (B) HD: 8.33*10^6 cells/mL) and were subjected to either continuous Dynamic Compression (DC) or Intermittent Dynamic Compression (IDC) at 1Hz. Relative gene expression levels of chondrogenic (SOX9, ACAN, COL2A1) and hypertrophic (RUNX2, COL10A1) markers were determined using the $2^{-\Delta\Delta Ct}$ method. All values were normalized to the housekeeping gene B2M. All data represented as mean ± SD; n=4; *p≤0.05, **p≤0.01, ****p≤0.0001.

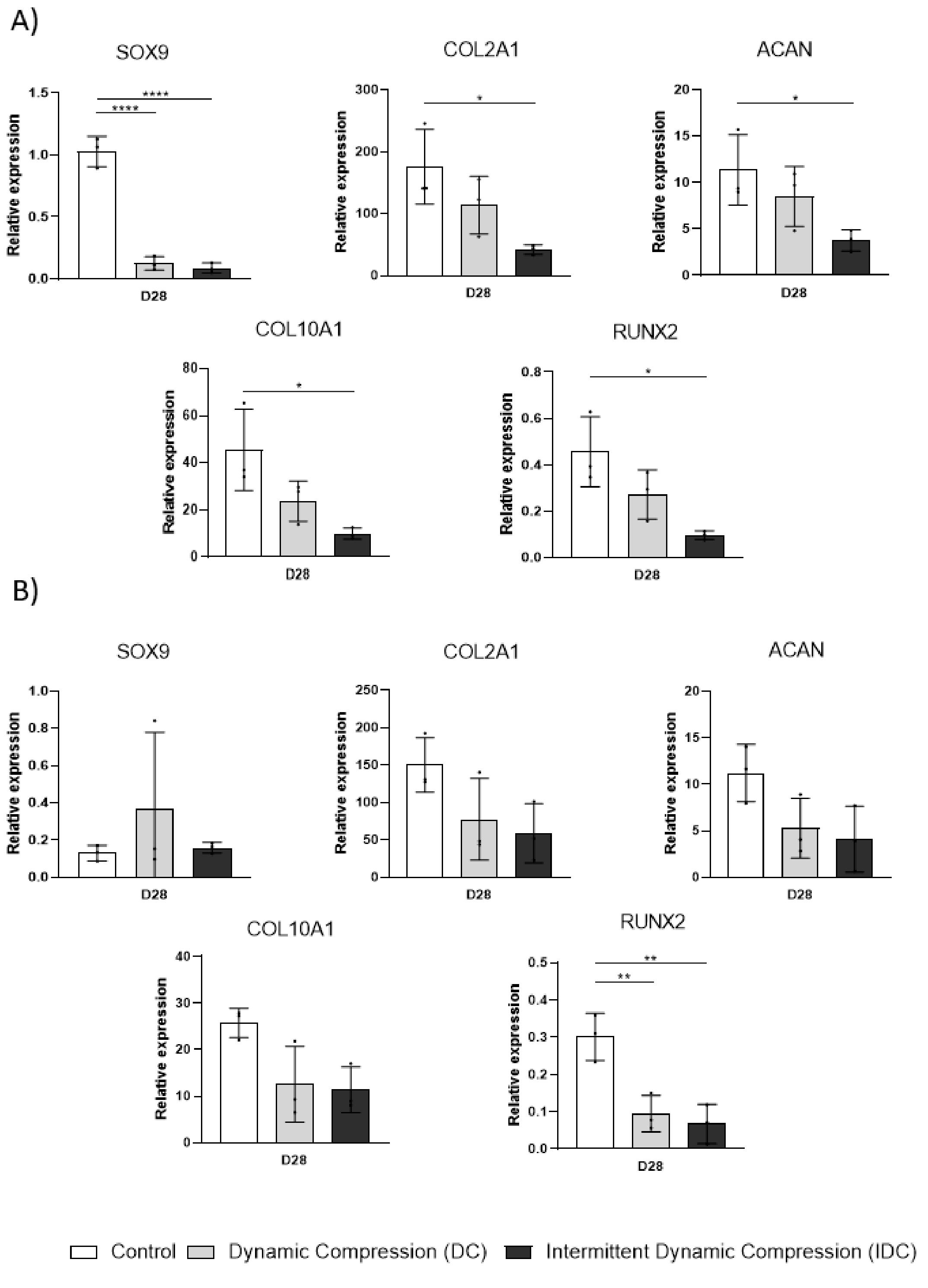

Supplementary figure S4: Human MSCs were encapsulated in 2.5 % fibrin hydrogels at two cell densities and were cultured for three weeks in presence of TGF-β3. In the fourth week, TGF-β3 was withdrawn and mechanical compression was applied. Continuous Dynamic Compression (DC) or Intermittent Dynamic Compression (IDC) were applied at 1 Hz. A) Low density (LD): $4.17*10^6$ cells/mL. B) High density (HD): $8.33*10^6$ cells/mL. Relative gene expression levels of chondrogenic (SOX9, ACAN, COL2A1) and hypertrophic (RUNX2, COL10A1) were determined using $2^{-\Delta Ct}$ method. All values were normalized to the housekeeping gene B2M. All data represented as mean ± SD; n=4; *p≤0.05; **p≤0.01; ***p≤0.001.
